# Prognostic Factors of Survival for High-Grade Neuroendocrine Neoplasia of the Bladder: A SEER Database Analysis

**DOI:** 10.3390/curroncol29080461

**Published:** 2022-08-18

**Authors:** Veronica Mollica, Francesco Massari, Elisa Andrini, Matteo Rosellini, Andrea Marchetti, Giacomo Nuvola, Elisa Tassinari, Giuseppe Lamberti, Davide Campana

**Affiliations:** 1 Department of Experimental, Diagnostic and Specialty Medicine, University of Bologna, 40138 Bologna, Italy; 2 Medical Oncology, IRCCS Azienda Ospedaliero-Universitaria di Bologna, 40138 Bologna, Italy

**Keywords:** bladder carcinoma, LCNEC, MiNEN, NEC, NEN, neuroendocrine, SCNEC, SEER

## Abstract

*Background:* High-grade neuroendocrine carcinoma (NEC) is a rare and aggressive variant of bladder cancer. Considering its rarity, its therapeutic management is challenging and not standardized. *Methods:* We analyzed data extracted from the Surveillance, Epidemiology, and End Results (SEER) registry to evaluate prognostic factors for high-grade NEC of the bladder. *Results:* We extracted data on 1134 patients: 77.6% were small cell NEC, 14.6% were NEC, 5.5% were mixed neuro-endocrine non-neuroendocrine neoplasia, and 2.3% were large cell NEC. The stage at diagnosis was localized for 45% of patients, lymph nodal disease (N+M0) for 9.2% of patients, and metastatic disease for 26.1% of patients. The median overall survival (OS) was 12 months. Multivariate analysis detected that factors associated with worse OS were age being >72 years old (HR 1.94), lymph nodal involvement (HR 2.01), metastatic disease (HR 2.04), and the size of the primary tumor being >44.5 mm (HR 1.80). In the N0M0 populations, the size of the primary tumor being <44.5 mm, age being <72 years old, and major surgery were independently associated with a lower risk of death. In the N+M0 group, the size of the primary lesion was the only factor to retain an association with OS. *Conclusions:* Our SEER database analysis evidenced prognostic factors for high-grade NEC of the bladder that are of pivotal relevance to guide treatment and the decision-making process.

## 1. Introduction

Bladder cancer is a quite common neoplasia, with about 81,000 estimated new cases in 2022 in the United States [1]. A great variety of different histologic morphologies can be found in the spectrum of bladder cancer: pure urothelial carcinoma (UC), UC with squamous or glandular differentiation, sarcomatoid, micropapillary, nested, plasmacytoid, and neuroendocrine carcinoma (NEC) [2,3]. The latter is a rare histology and can be divided based on differentiation and grade into small cell NEC (SCNEC), large cell NEC (LCNEC), and mixed neuroendocrine non-neuroendocrine neoplasia (MiNEN) [4]. The most acclaimed hypothesis on the origin of neuroendocrine tumors of the bladder is the presence of a multipotent undifferentiated stem cell in the urothelium, from which originate both the neuroendocrine and the urothelial components [5].

The most aggressive variant is SCNEC, which accounts for about 1% of urinary bladder tumors [6,7]. It is more frequent in the male sex and commonly affects patients older than 50 years. Histologically and genomically, SCNEC is distinguished from UC, which is the most frequent type of bladder cancer, by the presence of neuroendocrine differentiation and immunohistochemical characteristics [8,9]. Neuron-specific enolase (NSE), chromogranin A, and synaptophysin, which are markers of neuroendocrine differentiation, are often expressed in SCNEC of the bladder [10]. Other immunohistochemistry markers can help to differentiate SCNEC and UC: the expression of p16 associated with no expression of p63 and CK20 is typical of SCNEC of the bladder, while p63 and CK20 are frequently expressed in high-grade UC, with either negative or positive p16 [11]. SCNEC often presents loss of TP53 and RB1, TERT promoter mutations, and high somatic mutational burden [12].

The other neuroendocrine forms are even rarer, and data are very limited. A correct histologic diagnosis is often challenging and requires a high level of expertise by the pathologist; thus, some of the divergent neuroendocrine histology reported could be misdiagnosed SCNEC. Consequently, it is strongly recommended to address these cases to neuroendocrine neoplasia referral centers to increase the quality of diagnostic and therapeutic management [13,14,15].

The prognosis of NEC histology is dismal and overall worse than its UC counterpart. Different series reported scant survival, with a median overall survival (OS) of 11 months, with significant differences depending on the stage at diagnosis, and 5-year survival rates ranging from around 60% for stage II disease to 10% for stage IV tumors [16,17,18].

The majority of cases (about 60%) of NEC of the bladder are diagnosed in an advanced stage, which is correlated with a worse prognosis. Neoadjuvant chemotherapy followed by radical surgery is the best treatment option to increase OS and disease-free survival (DFS) in non-metastatic NEC [8,19,20,21]. The standard chemotherapy treatment in a perioperative setting and in an advanced stage is a platinum-based regimen, in particular cisplatin plus etoposide [19].

Given the rarity of this disease and the consequent limited and discordant data derived from the available studies, which were mostly retrospective, we extracted data from the Surveillance, Epidemiology, and End Results (SEER) registry with the aim to better assess prognostic factors of survival that could guide the optimal treatment strategy for high-grade neuroendocrine neoplasia of the bladder.

## 2. Materials and Methods

We extracted the data of patients with histologically confirmed neuroendocrine carcinoma of the bladder from the SEER database, registered from 1975 to 2018. We used the SEER*stat software (https://seer.cancer.gov, accessed on 25 May 2022) and identified patients with NEC histology of the bladder and with localized or advanced/metastatic disease. The histology diagnosis code 8002,8040-8045,8154,8240-8242,8244-8246,8249 (ICD-O-3) was used to select cases from the “Incidence—SEER Research Data, 9 Registries, Nov 2020 Sub (1975–2018)—Linked To County Attributes—Time Dependent (1990–2018) Income/Rurality, 1969–2019 Counties, National Cancer Institute, DCCPS, Surveillance Research Program, released April 2021, based on the November 2020 submission.

Permission to access the SEER database was granted on 19 March 2020 with authorization number 22375-Nov 2020.

We collected epidemiological, clinical, and pathological data from the registry. We analyzed age as a continuous variable, dichotomized as a median value. We identified the median age of the population as cut-off. As for histology, we included only patients with high-grade neuroendocrine carcinoma and we excluded neuroendocrine tumors (NET) G1 and G2. Stage was divided into localized, lymph node involvement (N+), distant metastases (M+), and not available (NA). Considering that TNM staging varied over time, we analyzed the TNM staging of each individual patient according to the classification of the year of diagnosis of each patient, and we evaluated it in order to make it uniform among patients. Data on the extent of lymphadenectomy and number of positive lymph nodes were not available.

The following sites of distant metastasis were extracted: bone, liver, lung, central nervous system (CNS). Surgery of the primary lesion was categorized as “minor” (including transurethral resection, partial cystectomy) or “major” (including total cystectomy, pelvic exenteration). It must be underlined that in women, radical cystectomy consists of anterior pelvic exenteration (removal of urethra, lower part of the ureters, uterus, cervix, vagina, and bladder). The number of negative (N0) or positive (N+) lymph nodes was extracted and compared with the number of total lymph nodes removed. Not reported lymph node status was addressed as NA.

### Statistical Analysis

OS was the primary endpoint of the analysis and was defined as the time from diagnosis to death by any cause. OS was estimated by the Kaplan–Meier method and was reported in months (95% confidence interval—CI). Results were compared with the log-rank method. Predictive risk factors for OS were analyzed by univariate and multivariate analysis using the Cox proportional hazards method and expressed as hazard ratios (HR). The multivariate model was fitted using the backward stepwise method after including all variables. In the multivariate analysis, we included all variables of the univariate analysis, both statistically significant and not. The area under the receiver-operating characteristic (ROC) curve was evaluated to determine the best prognostic cut-off value for the size of the primary lesion in millimeters (mm). The *p* value was considered significant when <0.05. The statistical analysis was carried out using IBM—SPSS Statistics v. 22.

## 3. Results

### 3.1. Overall Patients Characteristics

Records from 1134 patients matching the inclusion criteria were extracted from the SEER registry and included in the analysis. Patient characteristics of the whole population are summarized in Table 1. The majority of patients, as expected, were male (*n* = 903, 79.6%). Histologically, 77.6% (*n* = 880) were SCNEC, 14.6% (*n* = 166) were NEC, 5.5% (*n* = 62) were MiNEN, and 2.3% (*n* = 26) were LCNEC. With regards to stage at diagnosis, 45% (*n* = 510) of patients had localized disease, 9.2% (*n* = 104) had lymph nodal disease, and 26.1% (*n* = 296) had metastatic disease, and in 19.7% (*n* = 224) the stage was NA. Surgical removal of the primary lesion was performed in 75.6% (*n* = 869) of patients. At 12 months from diagnosis, 49.4% (*n* = 560) were dead, 44.7% (*n* = 507) were alive, and 5.9% (*n* = 67) had a follow up shorter than 12 months.

### 3.2. Survival Analysis

**Figure 1:**
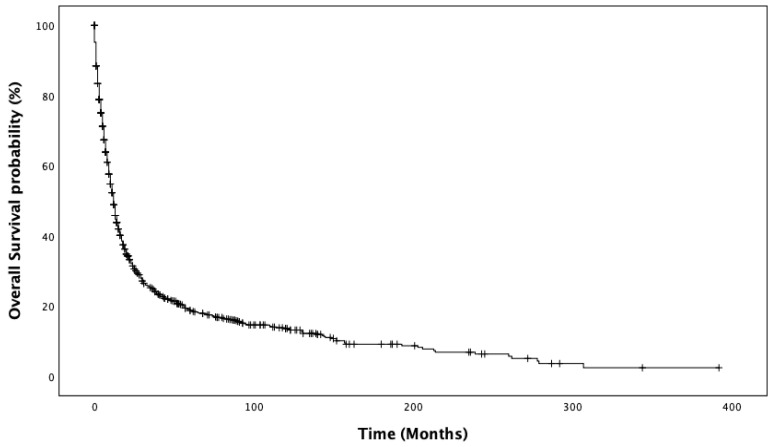
Kaplan–Meier estimates of overall survival.

In the entire population, the median OS was 12 months (95%CI 10.9–13.1 IC95%). The Kaplan–Meier estimates of overall survival are reported in Figure 1. Significant differences in the median OS were observed according to age (16 months, 95%CI 13.5–18.4, for patients <72 years old vs. 9 months, 95%CI 7.5–10.4, for those >72 years old, *p* < 0.001; Supplementary Figure S2), stage at diagnosis (20 months, 95%CI 15.4–24.5, for localized disease vs. 13 months, 95%CI 10.7–15.2, for N+ vs. 6 months, 95%CI 4.8–7.1, for M+, *p* < 0.001; Supplementary Figure S3), and surgery of the primary tumor conducted at 13 months, 95%CI 11.7–14.2 vs. not conducted at 4 months, 95%CI 2.7–5.2, *p* < 0.001; Supplementary Figure S4). For patients who underwent surgery, the extent of the surgical procedure was associated with a significant difference in OS (12 months for minor surgery, 95%CI 10.7–13.2 vs. 27 months, 95%CI 18.7–35.2 for major surgery, *p* < 0.001; Supplementary Figure S5). Significant differences in OS were also observed among patients with different metastatic sites: bone (*p* < 0.001), liver (*p* < 0.001), SNC (*p* = 0.008), and lung (*p* < 0.001) compared to those without metastatic involvement of those sites, respectively. No significant difference in OS was observed according to sex (*p* = 0.648) and histology (*p* = 0.148).

We calculated the best cut-off for the size of the primary tumor to predict survival at 12 months, for the 528 patients with available data, in an unbiased way by a ROC curve at 44.5 mm (Supplementary Figure S1), with 62.2% sensibility and 62.3% specificity.

### 3.3. Prognostic Factors in the Overall Population

Risk factors for OS are reported in Table 2. At univariate analysis, significant risk factors for worse survival were age being >72 years old (*p* < 0.001), lymph nodal involvement (*p* < 0.001), metastatic disease (*p* < 0.001), the size of the primary lesion being >44.5 mm (*p* < 0.001), bone (*p* < 0.001), liver (*p* < 0.001), lung (*p* < 0.001), SNC (*p* = 0.008), and minor surgery (*p* < 0.001). In multivariate analysis, the only factors confirmed to be independently associated with worse OS were ages which were >72 years old (HR 1.94, *p* < 0.001), lymph nodal involvement (HR 2.01, *p* < 0.001), metastatic disease (HR 2.04, *p* = 0.001), and the size of the primary lesion being >44.5 mm (HR 1.80, *p* < 0.001).

Furthermore, we assessed whether the size of the primary lesion was associated with the lymph nodal status. Using ROC curve analysis, the best cut-off of primary tumor size that correlated with positive lymph nodal status was 39.5 mm (74% sensibility, 55% specificity, AUC 0.595, standard error 0.032, *p* = 0.005).

### 3.4. Prognostic Factors for Patients with N0M0 Disease

Characteristics of patients with no lymph node involvement nor metastatic lesions (N0M0, *n* = 510) are reported in Table S1. The median OS for the N0M0 subgroup was 20 months (95%CI 15.4–24.5) and the Kaplan–Meier estimates of OS are reported in Supplementary Figure S6. Variables significantly associated with different OS were age (*p* < 0.001), the size of the primary tumor (*p* < 0.001), surgery (*p* < 0.001), and the extent of surgery (*p* < 0.001), while no significant difference was found according to sex (*p* = 0.816) and histology (*p* = 0.579). Multivariate analysis showed that the size of the primary tumor being <44.5 mm (HR 1.61, *p* < 0.001), age being <72 years old (HR 1.80, *p* < 0.001), and major surgery (HR 1.58, *p* = 0.021) were independently associated with a lower risk of death (reported in Table S2).

### 3.5. Prognostic Factors for Patients with N+M0 Disease

There are 104 patients with N+M0 disease whose characteristics are summarized in Table S3. In this population, the median OS was 13 months (95%CI 10.7–15.2). The Kaplan–Meier estimates of OS are reported in Supplementary Figure S7. Factors associated with OS were age (*p* = 0.018) and the size of the primary tumor (*p* = 0.027), while no difference was observed according to surgery (*p* = 0.090) and its extent (*p* = 0.147). Multivariate analysis showed that the size of the primary lesion was the only factor to retain its association with OS (HR 1.88, *p* = 0.029), while surgery did not (Table S4).

## 4. Discussion

Our population-based analysis is one of the largest studies in the literature and is based on a meaningful number of patients with the rare neuroendocrine histology of a bladder tumor, who were extracted from the SEER registry. Our study aims to assess the prognostic factors along with their survival repercussions that could guide the best treatment approach. We show several clinically meaningful findings. First, our results show that surgery, in particular major procedures intended as radical cystectomy or pelvic exenteration, are associated with better OS but only in patients with localized disease and no lymph node involvement while, in the case of positive lymph nodes, this benefit is lost. This result corroborates other data reported in the literature that underline the pivotal value of early diagnosis and radical treatment of patients with this aggressive disease. This result is similar to the standard of care management of patients with N0M0 UC of the bladder and small cell lung carcinoma, for which a radical approach is the cornerstone to achieve better survival outcomes [22,23]. In addition, we did not find a statistically significant difference in OS according to histology (*p* = 0.148). This may be because the majority of the included patients were SCNEC. This also suggests a common scant prognosis of high-grade histologies, as reported for neuroendocrine neoplasia arising in other organs.

Moreover, we confirmed the dismal survival outcome of these patients even with the increased expertise over the years in surgical and general clinical management. In fact, the median OS in the population was 12 months, concordant with data from other reports that showed a median OS for SCNEC patients (the majority of those included in our study and in the population included in other dedicated studies) ranging from 11 months [16,18] to 21 months in non-metastatic patients [24].

In addition, we showed that a large size of the primary tumor impacts OS, presumably due to the consequent higher rate of lymph nodal involvement, that remains one of the main risk factors for OS.

Our data were consistent with those of another SEER database analysis conducted by Koay et al. in 2011 [16] and of a single institution retrospective study on 38 patients led by Jung in 2016 [18], that both reported a median OS of 11 months.

Neoadjuvant chemotherapy is strongly recommended since, in several reports, it seems to be associated with the best outcomes in terms of OS, DFS, and pathologic downstaging [20,25]. A multicenter retrospective study led by a group of our institution on 51 cases of NEC of the bladder showed a beneficial trend in terms of relapse-free survival for radically resected patients that underwent perioperative chemotherapy compared with surgery alone [26]. Moreover, this analysis showed a trend toward better PFS in advanced patients treated with carboplatin and etoposide compared to other chemotherapy regimens. The pivotal role of neoadjuvant chemotherapy emerges from multiple studies, suggesting that a shrinkage of the primary lesion could result in an improved and more radical surgical approach, translating into improved patient survival outcomes. The role of neoadjuvant chemotherapy appears to be more relevant in patients without lymph nodal involvement [20], similarly to the urothelial counterpart.

Our study presents some limitations. First, these results should be treated with caution, considering that they derive from a retrospective registry. To limit potential bias, we restricted the analyzed population according to distinct factors that we could use to make patient groups homogeneous. Another limitation is the lack of potentially relevant confounding factors that could not be included due to missing data, such as performance status, chemotherapy treatment and regimen, and disease-/progression-free survival. Furthermore, we did not investigate the interaction between older age (>72 years), which could be correlated with a higher morbidity, and poor prognosis. We did not perform cancer-specific survival and relapse-free survival analysis due to a lack of data on the majority of patients. The lack of these factors could potentially influence prognosis, but it must be underlined that the use of nationwide registries is a pivotal strategy to aggregate data for rare neoplasia.

Indeed, one of the main strengths of our analysis is the use of a nationwide registry to recollect a wide population with a rare disease, for which only retrospective studies or small case series are available.

An interesting observation emerging from our study that should be underlined involves patients that did not receive surgery. In fact, for N+M0 patients, multivariate analysis did not show a significant benefit of surgery. Notably, in retrospective series that derive from real-world practice, patients referred for surgery are commonly the most fit in terms of performance status and comorbidities, which generally results in a positive benefit in statistical analysis. Our result does not corroborate this common finding. It can be hypothesized that, as emerged also for urothelial carcinoma of the bladder, lymph node involvement mostly requires a chemotherapy approach, and surgery loses the pivotal role that retains for localized disease with no nodal metastases, due to the aggressiveness of a bladder tumor in both histotypes. This supports the extremely important need for an early diagnosis and rapid treatment.

## 5. Conclusions

In conclusion, our SEER database analysis allowed us to individuate important prognostic factors for high-grade neuroendocrine carcinoma of the bladder. We evidenced that age being >72 years old, lymph nodal involvement, metastatic disease, and the size of the primary lesion being >44.5 mm are all factors independently associated with worse OS. Moreover, surgery of the primary tumor, particularly major surgery, is a pivotal therapeutic step to ensure the best treatment available for patients with no lymph node metastases. This finding suggests the need for an early diagnosis to increase patients’ survival outcomes.

## Figures and Tables

**Table 1:** Patient characteristics.

**Age**	Median	72 years
	≤72 years	46.4%
	>72 years	53.6%
**Sex**	Female	20.4% (*n* = 231)
	Male	79.6% (*n* = 903)
**Stage**	Localized	45% (*n* = 510)
	Lymph node metastases	9.2% (*n* = 104)
	Distant metastases	26.1% (*n* = 296)
	NA	19.7% (*n* = 224)
**Histology**	SCNEC	77.6% (*n* = 880)
	NEC	14.6% (*n* = 166)
	MiNEN	5.5% (*n* = 62)
	LCNEC	2.3% (*n* = 26)
**Site of metastases**	Bone	74/607 evaluated pts
	CNS	10/605 evaluated pts
	Liver	92/607 evaluated pts
	Lung	32/608 evaluated pts
**Type of surgery**	Minor	61.3% (*n* = 695)
	Major	15.3% (*n* = 174)
	NA	23.4% (*n* = 265)
**Status at 12 months**	Dead	49.4% (*n* = 560)
	Alive	44.7% (*n* = 507)
	Follow-up < 12 months	5.9% (*n* = 67)

Abbreviations: N, number of patients. NA, not available. SCNEC, small cell neuroendocrine carcinoma. LCNEC, large cell neuroendocrine carcinoma. MiNEN, mixed neuroendocrine non-neuroendocrine neoplasia. NEC, neuroendocrine carcinoma. CNS, central nervous system.

**Table 2:** Univariate and multivariate Cox proportional hazard models for the risk of death. Significant *p*-values are highlighted in bold.

	Univariate			Multivariate		
	HR	95%CI	*p*	HR	95%CI	*p*
Female sex	1.04	0.88–1.22	0.656	-	-	-
Age > 72aa	1.65	1.44–1.88	<0.001	1.94	1.47–2.55	<0.001
Stage						
N0	1					
N+	1.70	1.34–2.16	<0.001	2.01	1.37–2.94	<0.001
M+	3.01	2.54–3.55	<0.001	2.04	1.32–3.15	0.001
Diameter > 44.5 mm	1.65	1.34–2.03	<0.001	1.80	1.37–2.36	<0.001
Bone metastases	2.43	1.85–3.20	<0.001			NSS
Lung metastases	2.32	1.60–3.38	<0.001	1.85	1.03–3.33	0.38
SNC metastases	2.44	1.21–4.93	0.008			NSS
Liver metastases	2.75	2.16–3.51	<0.001	1.70	1.05–2.75	0.31
Minor surgery	1.98	1.60–2.45	<0.001	1.60	1.09–2.34	0.15

Abbreviations: HR, hazard ratio; 95%CI, 95% confidence interval; NSS, not statistically significant.

## Data Availability

These data were derived from the SEER cancer database available in the public domain: https://seer.cancer.gov. The data that support the findings of this study are available from the corresponding author upon reasonable request.

## Supplementary Materials

The following supporting information can be downloaded at: https://www.mdpi.com/article/10.3390/curroncol29080461/s1. Table S1: Patients with no lymph node involvement nor metastatic lesions (N0M0) characteristics. Table S2: Univariate and multivariate Cox proportional hazard models for the risk of death. Table S3: Patients with lymph node involvement and no metastatic lesions (N+M0) characteristics. Table S4: Univariate and multivariate Cox proportional hazard models for the risk of death. Figure S1: Receiving-Operator Characteristics (ROC) curve used to determine the best cut-off of size (≤44.5 mm or >44.5 mm). Figure S2: Kaplan–Meier estimates of overall survival according to age (≤2 or >72 years old). Figure S3: Kaplan–Meier estimates of overall survival according to stage (localized, lymph node or distant metastases). Figure S4: Kaplan–Meier estimates of overall survival according to surgery performed or not. Figure S5: Kaplan–Meier estimates of overall survival according to extension of surgery (major or minor). Figure S6: Kaplan–Meier estimates of overall survival of N0M0 patients. Figure S7: Kaplan–Meier estimates of overall survival of N+M0 patients.
