# Supplementary material for: Prognostic Factors of Survival for High-Grade Neuroendocrine Neoplasia of the Bladder: A SEER Database Analysis
Source: Curr Oncol. 2022 Aug 18;29(8):5846–54. doi: 10.3390/curroncol29080461 (PMC9406377; doi:10.3390/curroncol29080461)
Supplement: Supplementary file 1 [file curroncol-29-00461-s001.zip › curroncol-1835645-supplementary.pdf]

Supplementary Materials: Prognostic Factors of Survival for High-Grade Neuroendocrine Neoplasia of the Bladder: A SEER Database Analysis

Veronica Mollica, Francesco Massari, Elisa Andrini, Matteo Rosellini, Andrea Marchetti, Giacomo Nuvola, Elisa Tassinari, Giuseppe Lamberti and Davide Campana

Table S1. Patients with no lymph node involvement nor metastatic lesions (N0M0) characteristics (total number of patients = 510).

**Age**	≤72 years	43.7% (*n* = 223)
	>72 years	56.3% (*n* = 287)
**Sex**	Female	19.8% (*n* = 101)
	Male	80.2% (*n* = 409)
**Histology**	SCNEC	75.3% (*n* = 384)
	NEC	13.5% (*n* = 69)
	MiNEN	7.8% (*n* = 40)
	LCNEC	3.3% (*n* = 17)
**Type of surgery**	Minor	80% (*n* = 391)
	Major	20% (*n* = 98)
	NA	23.4% (*n* = 265)
**Status at 12 months**	Dead	40.3% (*n* = 190)
	Alive	59.7% (*n* = 282)

Abbreviations: N, number of patients. NA, not available. SCNEC, small cell neuroendocrine carcinoma. LCNEC, large cell neuroendocrine carcinoma. MiNEN, mixed neuroendocrine non-neuroendocrine neoplasia. NEC, neuroendocrine carcinoma.

Table S2. Univariate and multivariate Cox proportional hazard models for the risk of death. Significant p-values are highlighted in bold.

	**Univariate**			**Multivariate**		
	**HR**	**CI95%**	***p***	**HR**	**CI95%**	***p***
Female sex	1.03	0.80-1.35	0.818	-	-	-
Age >72 years	1.96	1.57-2.46	<0.001	1.80	1.32-2.47	<0.001
Diameter >44.5 mm	1.64	1.23-2.17	<0.001	1.61	1.21-2.15	0.001
Minor surgery	2.32	1.68-3.19	<0.001	1.58	1.07-2.35	0.021

Abbreviations: HR, hazard ratio; 95%CI, 95% confidence interval.

Table S3. Patients with lymph node involvement and no metastatic lesions (N+M0) characteristics (total number of patients = 104).

**Age**	≤72 years	49.0% (*n* = 51)
	>72 years	51.0% (*n* = 53)
**Sex**	Female	18.3% (*n* = 19)
	Male	81.7% (*n* = 85)
**Histology**	SCNEC	81.7% (*n* = 85)
	NEC	6.7% (*n* = 7)
	MiNEN	10.6% (*n* = 11)
	LCNEC	1% (*n* = 1)
**Type of surgery**	Minor	52.6% (*n* = 51)
	Major	47.4% (*n* = 46)
	Dead	51.1% (*n* = 47)

**Status at 12 months**	Alive	48.9% (*n* = 45)

Abbreviations: SCNEC, small cell neuroendocrine carcinoma. LCNEC, large cell neuroendocrine carcinoma. MiNEN, mixed neuroendocrine non-neuroendocrine neoplasia. NEC, neuroendocrine carcinoma.

Table S4. Univariate and multivariate Cox proportional hazard models for the risk of death. Significant p-values are highlighted in bold.

	Univariate			***Multivariate***		
	**HR**	**CI 95%**	***p***	**HR**	**CI 95%**	***p***
Female sex	1.05	0.58-1.90	0.875			-
Age >72 years	1.66	1.07-2.56	0.022			NSS
Diameter >44.5 mm	1.85	1.05-3.25	**0.033**	1.88	1.07-3.32	0.029
Surgery performed	0.52	0.24-1.14	0.105			-
Minor surgery	1.38	0.88-2.17	0.159			-

Abbreviations: HR, hazard ratio; 95%CI, 95% confidence interval; NSS, not statistically significant.

NSS, not statistically significant.

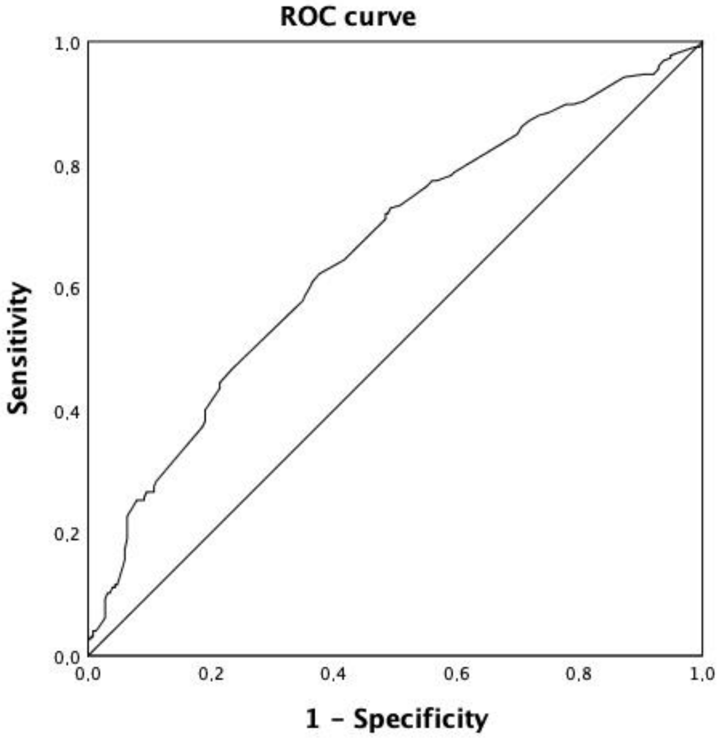

Figure S1. Receiving-Operator Characteristics (ROC) curve used to determine the best cut-off of diameter (≤44.5 mm or >44.5 mm).

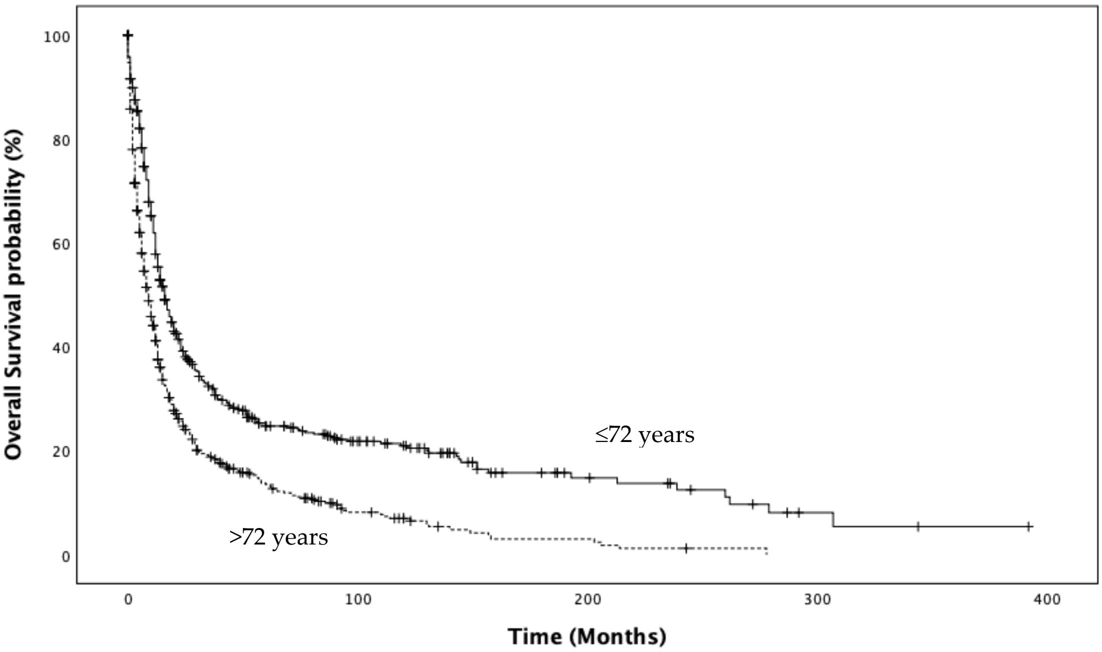

Figure S2. Kaplan-Meier estimates of overall survival according to age (72 or >72 years old).

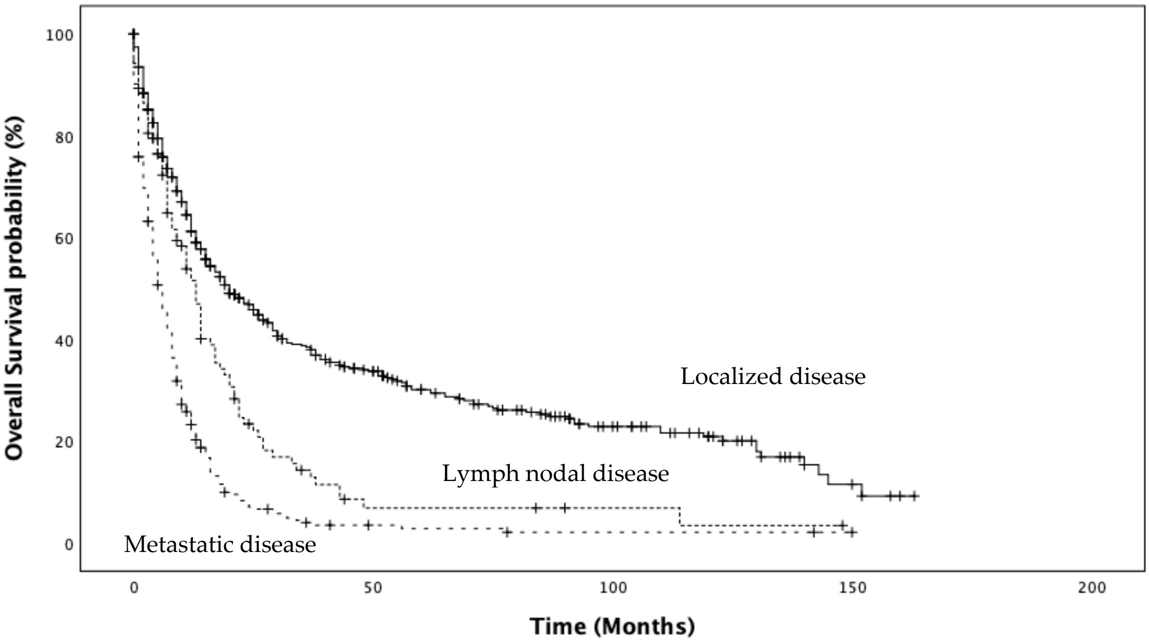

Figure S3. Kaplan-Meier estimates of overall survival according to stage (localized, lymph node or distant metastases).

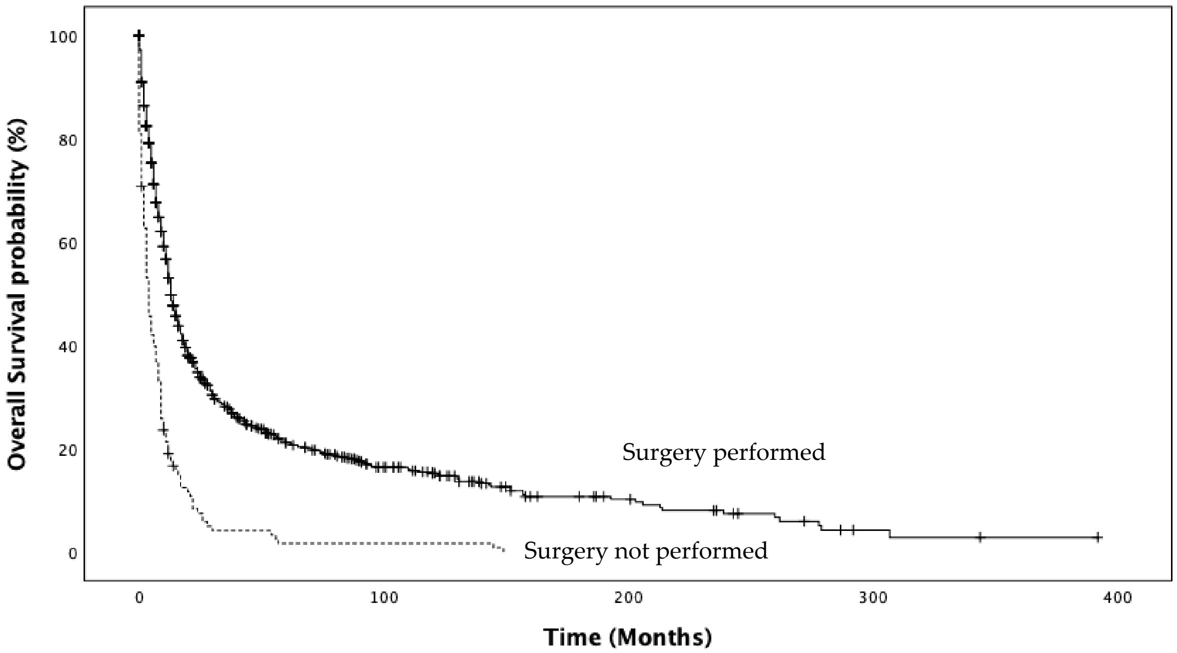

Figure S4. Kaplan-Meier estimates of overall survival according to surgery performed or not.

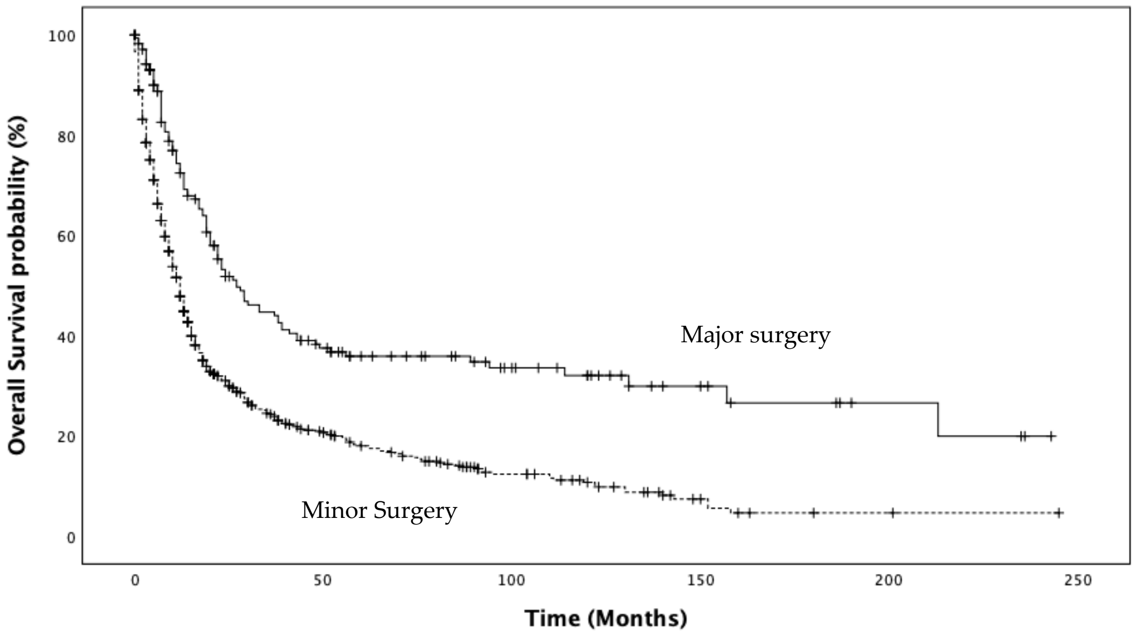

Figure S5. Kaplan-Meier estimates of overall survival according to extension of surgery (major or minor).

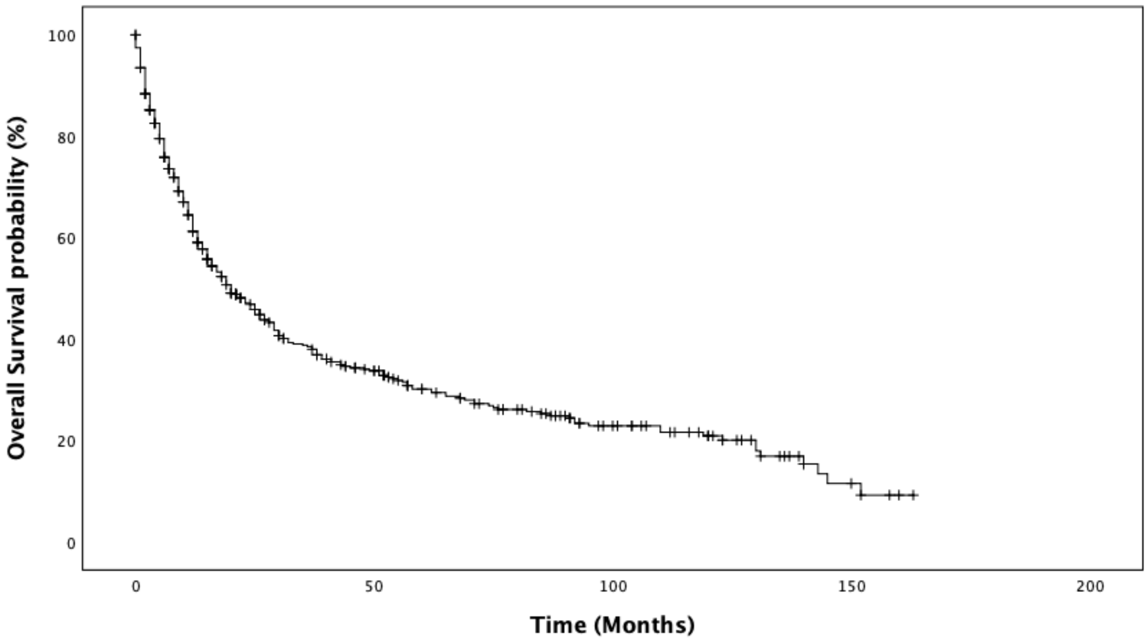

Figure S6. Kaplan-Meier estimates of overall survival of N0M0 patients.

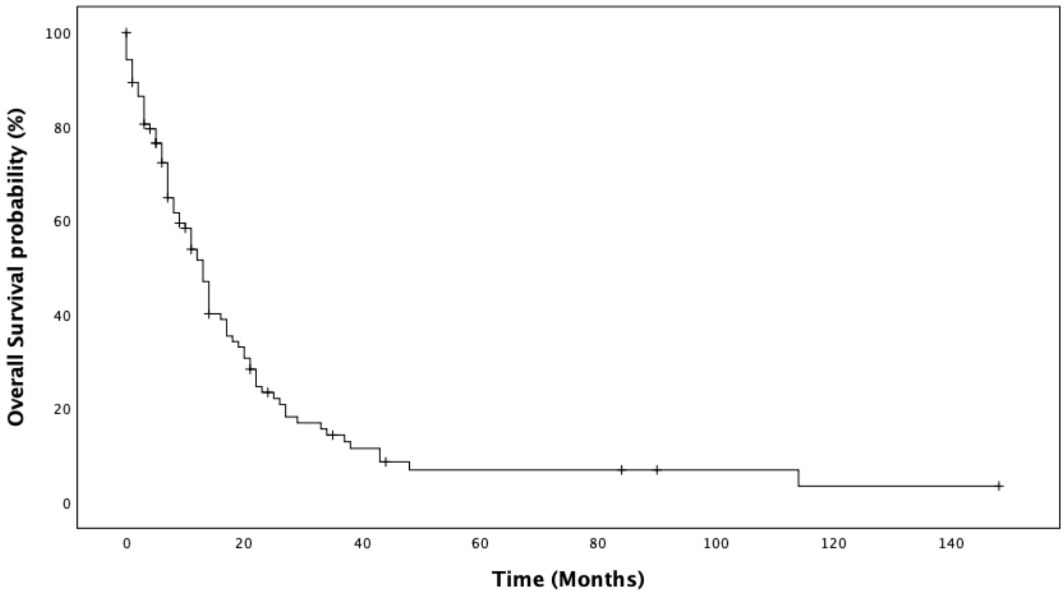

Figure S7. Kaplan-Meier estimates of overall survival of N+M0 patients.
